# An Aroma Precursor‐Based Approach to Improving the Sensory Quality of Thermally Treated Watermelon Juice

**DOI:** 10.1002/fsn3.70342

**Published:** 2025-06-13

**Authors:** Burcu Dundar Kirit, Erdal Ağçam, Asiye Akyildiz

**Affiliations:** ^1^ Department of Food Engineering, Faculty of Engineering Çukurova University Adana Turkey

**Keywords:** amino acid, aroma compounds, fatty acid, lycopene, optimization, watermelon juice pasteurization

## Abstract

Watermelon juice (WJ) is not widely consumed commercially in most countries due to the change in its sensory properties after thermal treatment, despite its attractive color and high lycopene content. To reveal the effect of heat on WJ, the sensory properties, aroma compounds, amino acids, fatty acids, hydroxymethyl furfural (HMF), lycopene content, and microbiological load analyses were carried out. Among them, HMF‐(−0.576), leucine‐(0.500), lycopene‐(0.539), nonenal‐(0.524), and nonadienol‐(0.506) were correlated with the aroma and/or overall acceptance scores obtained during sensory evaluation. The mathematical models describing the changes in watermelon juice components as a function of thermal processing parameters were established and validated (error ≤ 8.7%). The optimum pasteurization conditions were determined to be 84°C and 1 min to obtain WJ (desirability: 0.844), which have a higher acceptability, better aroma, and sufficient microbial reduction. The concentrations of nonenal, hexanal, and 6‐methyl‐5‐hepten‐2‐one, 2‐decenal decreased with increasing temperature and duration of pasteurization, unlike those of dimethyl disulfide. The variations in 6‐methyl‐5‐hepten‐2‐one and 2‐decenal concentrations as a function of thermal treatment conditions exhibited similar trends. Leucine concentration was influenced to a greater extent by processing time compared to temperature. HMF formation in WJ was more pronounced than that of pasteurization time. Additionally, intensifying the pasteurization conditions led to an unfavorable change in all sensory attributes, contrasting with its effects on HMF and linolenic acid levels.

## Graphical abstract

Watermelon juice is not widely consumed commercially due to sensory changes after heat treatment, despite its appealing color and high lycopene content. Pasteurization at 84°C for 1 min was found optimal, preserving aroma compounds and related precursors, odor, and overall acceptability. Higher pasteurization conditions led to reduced sensory attributes and key aroma compounds, while increasing HMF and linolenic acid levels.

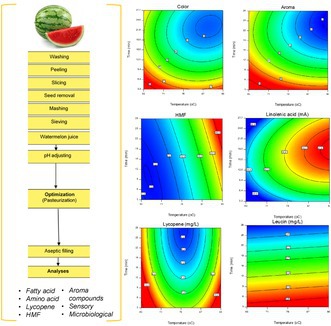

## 1 Introduction

Watermelon ( *Citrullus lanatus* ) juice (WJ) has a great potential due to its attractive color and content of bioactive compounds such as lycopene, vitamins, and amino acids; conversely, it can have undesirable changes in aroma after thermal treatment. Heat‐induced off‐flavors in WJ primarily arise from the formation of aldehydes, alcohols, and sulfur‐containing compounds during thermal processing. (*E*)‐2‐heptenal, decanal, octanol, diisopropyl disulfide, hexanol, (*E*)‐2‐decenal, and (*E*)‐2‐octenol were identified as off‐flavor aroma compounds in thermally treated WJ (Yang, Yang, et al. 2020a, 2020b). Although some non‐thermal processes have been shown to be a solution to the changes caused by heat treatment, it will take time for these processes to be applied on an industrial scale. It has been reported that pulsed UV light applications, which are among the non‐thermal treatments recently applied to WJs, provide sufficient microbial reduction (Pratap‐Singh and Mandal 2024), while ozone application can provide a 3‐log reduction in unclarified fruit juice (Lee et al. 2022). Additionally, when the effects of a pulsed electric field, high‐pressure processing, and thermal treatment, which ensure adequate microbial inactivation, on volatile compounds in WJ were evaluated, it has been reported that the thermally treated WJ was the most similar sample to the control (Aganovic et al. 2017). Hexanal, nonanal, and (*E*)‐2‐nonenal were considered to be the primary aroma compounds among the 47 compounds that were reported in WJ by solvent‐assisted flavor evaporation (SAFE) and solid‐phase microextraction (SPME) combined with gas chromatography‐olfactometry‐mass spectrometry (GC‐O‐MS) (Liu et al. 2018), while the key compounds linked to off‐flavor in thermally treated WJ included acetophenone, decanal, and (*E*)‐2‐decenal (Yang, Liu, et al. 2020). Due to their low aroma detection threshold, C6 and C9 aldehydes significantly influence the overall aroma of juice (Liu et al. 2018). The information required to monitor aroma release in food systems will be provided by an understanding of matrix factors that may affect the release of aroma compounds (Schober and Peterson 2004) or their formation/degradation. From this point of view, it is highly important to evaluate the effects of internal factors such as lycopene, fatty acid, amino acid, enzyme activity, and sugar contents, as well as the external factors such as temperature during treatment.

The impact of thermal treatment on the aroma of WJ was not thoroughly examined, nor was it correlated with variations in concentrations of aroma/aroma‐precursor compounds. This study aimed to reveal and model the effect of thermal treatment time and temperature on the aroma and selected compounds identified as potential aroma precursors. Furthermore, the thermal treatment conditions were optimized to obtain more preferable WJ in terms of aroma/sensory quality, addressing a key challenge associated with pasteurized WJ products.

## 2 Materials and Methods

### 2.1 Materials

Watermelons (*Starburst* cv.) were farmed uniformly at the same maturity from the Çukurova region, Adana, Turkey, in 2021. The fruits used in the present study had approximately the same color and size, and they were processed to nectar immediately after harvesting.

### 2.2 Methods

#### 2.2.1 WJ Production

The watermelons were washed, and the seeds were removed after the fruit flesh was separated from the rind. The flesh was crushed by using a blender (Waring Commercial Blender, USA), passed through a sieve with a pore diameter of 0.5 mm, and brought to a certain pH value (3.9) suitable for pasteurization by adding food‐grade citric acid. The production stages are detailed in File S1.

#### 2.2.2 Thermal Treatments

Pasteurization of the WJs was carried out using a continuous pasteurization system (File S2) that included a magnetic stirrer, peristaltic pump, stainless steel heat exchanger, and cooling chamber (Agcam et al. 2014).

#### 2.2.3 Experimental Design

The temperature and time required to cause the least change in quality characteristics were determined via an optimization study. Analyses within the scope of the study were applied to the samples obtained from these trial designs, and mathematical models were obtained from the results. The model adequacies were checked in terms of the values of *R* ^2^, adjusted *R* ^2^, and predicted *R* ^2^, as well as the no significant lack‐of‐fit value. While determining the heat treatment parameters applied in the optimization study by using *Design Expert* software (version 10.0; Stat‐Ease Inc., Minneapolis, USA), the independent variables, temperature and time intervals, were determined to be 60°C–98°C and 1–30 min, covering low‐temperature‐long time (LTLT) and high‐temperature‐short time (HTST) applications, respectively. The details are given in File S1, and the 17 different temperature–time combinations of applied thermal treatment with 5 central 2 factorials and 2 axial replicates are given in File S3.

#### 2.2.4 Validation

A validation study of the mathematical models created with the optimization method was carried out by applying the optimum conditions to WJ and determining the quality properties of the final product. The experimental results and the values estimated by the models were compared. The error levels were calculated by using the following equation (Equation 1):

(1) $$\text{Error}\;\left(\%\right)=\left|\frac{X_{t}-X_{a}}{X_{a}}\right|x100$$

In this equation, *X*_*a*_ defines the actual value, while *X*_*t*_ is the predicted value. When the error levels are < 10%, the obtained models are accepted to be successful at estimation (Dündar et al. 2019).

#### 2.2.5 Analyses

All the applied analysis methods were repeated at least three times.

##### 2.2.5.1 General Assays

The pH of the WJs was determined using a pH meter with a glass electrode (Mettler Toledo, USA). Ten milliliters of WJ was titrated with 0.1 N NaOH (Merck, Germany) to pH 8.1. The results were calculated as grams of citric acid per 100 mL of fresh weight. The soluble dry matter was measured with a portable refractometer (Kyoto Electronics RA‐130, Japan) at 20°C. The results are expressed as°Brix (Cemeroğlu 2007). The chromatograms obtained from HPLC and GC analyses are given in File S4.

##### 2.2.5.2 Free Amino Acid Composition

Amino acid analysis of the WJ samples was performed using high‐pressure liquid chromatography (HPLC) (Shimadzu, LC‐20AT, Japan) with modifications to the method applied by Sherovski et al. (2018). For amino acid analysis, 5 mL of sample was centrifuged by mixing after the addition of 1% HCl and passed through a paper filter. Equal amounts of Na_2_CO_3_ (0.4 M, pH = 9, Merck, Germany) and dansyl chloride (20 mg/L acetone (w/v), Sigma, Germany) were added to the extracts, which were subsequently kept at 70°C with stirring for 1 h. After passing through a PTFE filter (0.45 μm, Millipore, Germany), the solution was injected into the HPLC instrument. The flow rate was determined by 1 mL/min gradient elution, and the column temperature was 30°C. The mobile phases were 10 mM sodium acetate (dissolved in 5% acetonitrile, pH = 6.3; Merck, Germany) and acetonitrile.

##### 2.2.5.3 Fatty Acid Composition

To determine the fatty acid composition, oil extraction was performed from the samples, and after the fatty acid methyl esters were formed, analyses were performed via an Agilent GC–MS/MS (7890B GC‐7010B MS) instrument with an autosampler (Gerstel, Germany) equipped with a flame ionization detector and a capillary Agilent J&W DB‐WAX column (60 m × 0.25 μm × 0.25 μm) (Garde‐Cerdán et al. 2007). The samples were vortexed by adding n‐hexane and 2 N methanolic KOH (Merck, Germany) to extract oil. After waiting for a clear separation of the phases, the upper phase containing the methyl esters was removed with the help of an injector (1 μL) and injected into the gas chromatograph (AOCS, 1989). Garde‐Cerdán et al. (2007) applied chromatography conditions in the analysis. The oven temperature was held at 50°C for 1 min, then increased to 200°C at 25°C/min, and after waiting for 10 min, it was increased to 230°C at 3°C/min and held at this temperature for 18 min. The injector and detector temperatures were set at 250°C and 300°C. The sample amount was determined as 1 μL, the flow rate of the carrier gas (helium) was determined as 1 mL/min, and the split ratio was determined as 1:40. Fatty acids were identified by comparing the retention times of fatty acid methyl esters (FAME) with those of the standard 37‐component FAME mixture.

##### 2.2.5.4 Lycopene

For lycopene analysis of the samples, an HPLC method suggested by Meléndez‐Martínez et al. (2007) was used. First, a 2 g sample is mixed with 25 mL of extraction solvent (hexane/acetone/methanol, 50/25/25 ratio (v/v/v), 0.1% BHT (w/v)). The mixture is stirred thoroughly and then centrifuged (6000 rpm, 10 min, 4°C). The clear supernatant is collected and washed four times with 15 mL of distilled water. Next, saponification is performed by adding 15 mL of 10% KOH (w/v) and incubating under nitrogen gas for 1 h. The saponification reaction is terminated by adding 10% NaCl (w/v). The mixture is washed again four times with 15 mL of distilled water. The hexane phase is evaporated using a rotary evaporator at 35°C. The residue is dissolved in 2 mL of an acetone/methanol solution (1/2 ratio (v/v), containing 0.1% BHT (w/v)). The solution was passed through a PTFE filter (0.45 μm, Millipore, Germany), and injected into the HPLC device (Shimadzu, Japan). MeOH (A) and MTBE (B) (MTBE and MeOH containing 0.1% BHT (w/v); Merck, Germany) were used to form the mobile phase at a 1 mL/min flow rate (gradient flow) in a ProntoSIL (Bischoff Chromatography, Germany) C30 column at 20°C. The injection volume was determined to be 50 μL, and the elution time was 65 min. The calibration curves prepared with the help of the certified standard substance were used to calculate the concentration equivalent of the detected peak areas.

##### 2.2.5.5 Hydroxymethyl Furfural

Determination of hydroxymethyl furfural (HMF) and furfural in WJ samples was performed using HPLC (Gökmen and Acar 1999). The HPLC procedure was performed according to the methods of Ağçam and Akyıldız (2014). Chromatographic conditions were as follows: isocratic elution with a mobile phase consisting of methanol/water/acetic acid (20/79/1, v/v/v), a flow rate of 0.5 mL/min, an injection volume of 20 μL, and a total elution time of 15 min. A C18 column (ACE, 5 μm 250 × 4.6 mm) at 30°C and a photodiode detector (PDA) at 285 nm were used. HMF identification was based on the retention times and UV spectra of the injected standard compounds. The results were expressed in micrograms per liter (μg/L).

##### 2.2.5.6 Aroma Compounds

Aroma compounds, which are reported to be responsible for off‐flavor in WJs, can be extracted increasingly quickly via the solid‐phase microemulsion (SPME) method compared to the liquid–liquid (SPE) and solvent‐assisted aroma extraction (SAFE) methods (Yang, Yang, et al. 2020a, 2020b; Zhou et al. 2018). Therefore, the SPME method was applied in this study. WJ (10 mL) aroma compounds in the headspace of a 20 mL vial were extracted using divinylbenzene/carboxen/polydimethylsiloxane fibers (1 cm–50/30 μm). 2‐Methyl‐3‐heptanone (0.816 μg/μL; Sigma, Germany) was used as an internal standard and was added to WJ before extraction. After the equilibrium state was achieved by waiting for 15 min at 60°C, the fiber of the SPME device was positioned 1 cm above the liquid surface for 30 min. Gas chromatography (GC: Agilent 7890B—flame ionization detector (FID)) and a polar DB‐WAX column (60 m × 0.25 mm × 0.25 μm; J&W Scientific‐Folsom, USA) were used for the quantification of aroma compounds. The injection temperature was 250°C, the column temperature was increased at 40°C for 4 min, the temperature was increased by 3°C/min to 90°C, and the temperature was increased by 4°C/min to 130°C. After waiting at this temperature for 4 min, the temperature was increased by 5°C/min, after which the temperature was held constant for 8 min. The carrier gas was helium at a flow rate of 1.2 mL/min (Nuzzi et al. 2008). For the diagnosis of aroma substances, a Brand mass spectrometer (MS, Agilent 7010B) connected to a specified gas chromatograph was used. The ionization energy of the mass spectrometer was kept at 70 eV, the ion source temperature was 230°C, the quadrupole temperature was kept at 120°C, and scanning was carried out between 30 and 600 mass/charge (m/e). The diagnosis of the peaks was made by comparing the mass spectrum to the mass/charge in computer memory (NIST 14.0) (File S5).

##### 2.2.5.7 Sensory Analysis

The sensory properties of precooled (at 4°C) WJs in terms of taste, aroma, color, overall acceptance, and aroma characteristics were evaluated (Lawless and Heymann 2010). For the sensory evaluation of the samples, the graphic scale method was used for each feature (File S6). The panels were generated by 15 experienced panelists (7 females, 8 male) in the Sensory Analysis Laboratory of Çukurova University Food Engineering Department (Adana, Turkey). The panelists were selected based on availability, interest, and experience in sensory analysis. Before sensory evaluations, panelists were provided with standard aroma references representing key descriptors of WJ (cucumber, grass, fruity, floral, fatty, cooked, and green). The samples were in clear glasses labeled with randomly assigned 3‐digit numeric codes. The panelists first performed a rank‐rating evaluation of the samples' aroma using a structured 10‐cm line scale. The seven aroma descriptors used for each sample were as follows: cucumber, grass, fruity, floral, fatty, cooked, and green. Before the analysis, the same amount of WJ treated under the same conditions (repetitions) was mixed to reduce the sample number, to increase the ease of sensory analysis for the panelists, and to increase the reliability of the results.

##### 2.2.5.8 Statistical Analyses

The results of the quality analysis were subjected to variance analysis using SPSS 20.0 software (Chicago, IL, USA), and the significant differences were determined according to the analysis of variance (ANOVA) followed by Duncan's multiple comparison test. The Design‐Expert software (Stat‐Ease, USA) was used to perform statistical analysis and develop the mathematical models in the optimization study. The correlations between the “aroma” and “overall acceptance” scores and the compounds in WJ were determined by applying the Pearson correlation (*p* < 0.05) test in the same packet program.

## 3 Results and Discussion

The pH (3.81–3.97) and titratable acidity (0.303%–0.474% citric acid) results of the WJs are given in File S7, and the average total soluble dry matter content was determined to be 8.64% ± 0.56%. The pH values of all the samples are below 4.5, confirming the suitability of pasteurization for the WJs produced in this study.

### 3.1 Aroma Compounds

**FIGURE 1:**
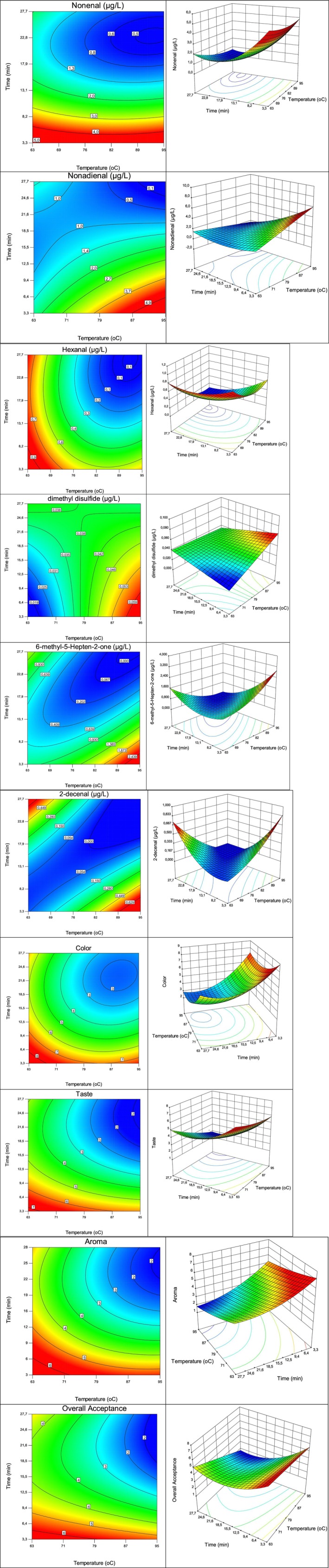
Effects of thermal treatment temperature and time on (*E*)‐2‐nonenal (*R* ^2^: 0.9511, adjusted *R* ^2^: 0.9289, predicted *R* ^2^: 0.8537); (*E*, *Z*)‐2,6‐nanodienal (*R* ^2^: 0.8883, adjusted *R* ^2^: 0.8375, predicted *R* ^2^: 0.7202); hexanal (*R* ^2^: 0.9159, adjusted *R* ^2^: 0.8776, predicted *R* ^2^: 0.7726); dimethyl disulfide (*R* ^2^: 0.2598, adjusted *R* ^2^: 0.0890, predicted *R* ^2^: −0.2278); 6‐methyl‐5‐hepten‐2‐one (*R* ^2^: 0.9223, adjusted *R* ^2^: 0.8869, predicted *R* ^2^: 0.7714); and (*E*)‐2‐decenal (*R* ^2^: 0.8750, adjusted *R* ^2^: 0.8182, predicted *R* ^2^: 0.6115).

A total of 38–41 aroma compounds, mostly aldehydes in addition to alcohols and esters, were observed in samples treated under different conditions (data not shown). The concentrations of all the aroma compounds were not given to avoid deviating from the purpose of the study because the most abundant volatiles are not necessarily the most important contributors to the aroma of WJ. 6‐methyl‐5‐hepten‐2‐one (green, grass), decanal (soap, orange peel, tallow), diisopropyl disulfide, dimethyl sulfide (cabbage, sulfur) and (*E*)‐2‐octenol were mentioned as aroma compounds responsible for off‐flavor, while (*E*)‐2‐nonenal (cucumber), (*E*, *Z*)‐2,6‐nonadienal, (*Z*, *Z*)‐3,6‐nonadienal, (*Z*)‐6‐nonen‐1‐ol, hexanal, (*E*)‐2‐hexenal, nonanal, (*E*)‐2‐nonenal, (*Z*)‐nonen‐1‐ol, (*E*)‐2‐octenal, 1‐nonanol, and (*Z*)‐3‐nonen‐1‐ol were related to the characteristic aroma of WJ (Aboshi et al. 2020; Yang, Yang, et al. 2020a, 2020b). Among them, (*E*)‐2‐nonenal, (*E*, *Z*)‐2,6‐nonadienal, 2‐decenal (tallow, orange), dimethyl disulfide (cooked, potato, onion, cabbage, putrid), hexanal, and 6‐methyl‐5‐hepten‐2‐one were identified and quantified in this study. The changes in the concentrations of these compounds with respect to the thermal treatment conditions are shown in Figure 1. No significant correlation was detected between the aroma compounds reported to be responsible for off‐flavor compounds in the literature and the aroma scores obtained by sensory analysis.

The concentration of nonenal was greater in the WJs thermally treated for a short time, and it decreased with increasing time. However, the temperature has no significant effect on the nonenal concentration after thermal treatment for a certain time. Similar results were also obtained for the (*E*, *Z*)‐2,6‐nonadienal concentration of WJ. The temperature had no effect on the nonadienal concentration after longer than ~18 min. On the other hand, a thermal treatment shorter than 13 min had an increasing effect on the nonadienal concentration. Among the studied temperatures and time intervals, the effect of temperature on the hexanal concentration was more significant than the effect of time, but the change pattern was similar to that of the nonenal temperature.

**FIGURE 2:**
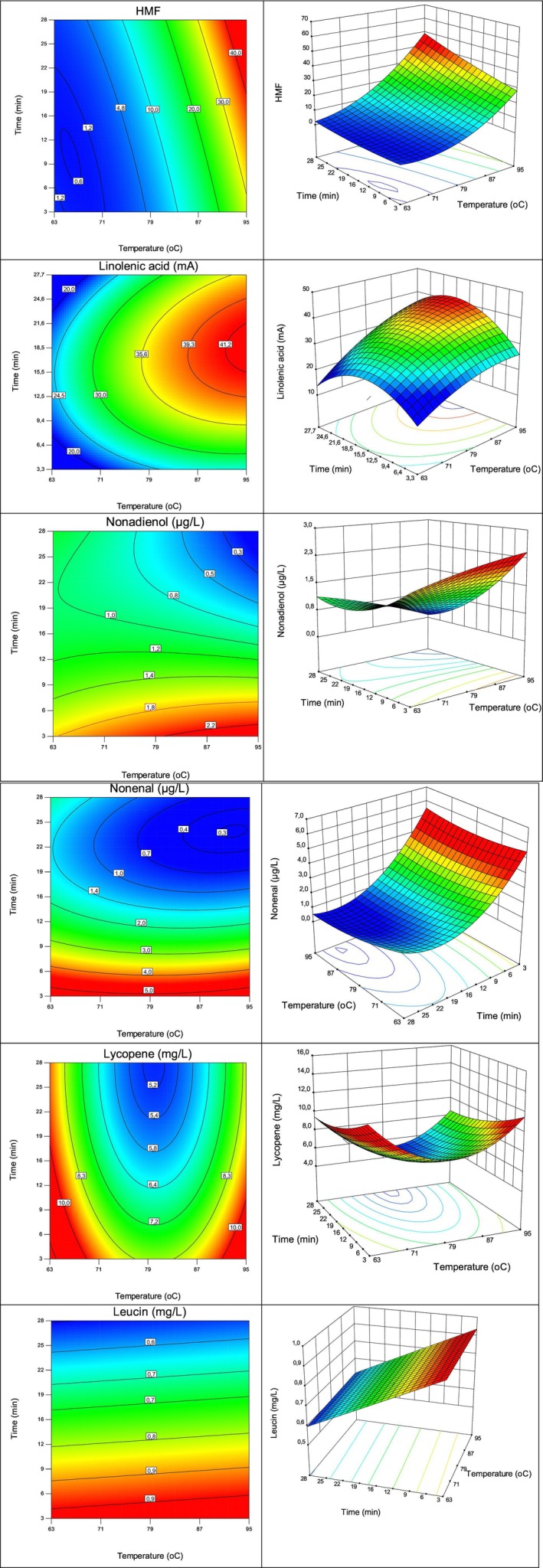
The effect of thermal treatment temperature and time on hydroxymethyl furfural (HMF), linolenic acid, (*E*,*Z*)‐3,6‐nonadienol, (*E*)‐2‐nonenal, lycopene, and leucine.

Although dimethyl disulfide, 6‐methyl‐5‐hepten‐2‐one, and 2‐decenal are related to the off‐flavor of WJ, their changes with respect to temperature are not similar. The main common increase observed for both of these genes was in the high‐temperature treatment group (Figure 2). The obtained results also showed that 2‐decenal increased with increasing temperature or duration of pasteurization but not with either temperature or duration. (*E*)‐2‐Decenal can be used as a marker of oxidation and is generated by oleic acid (Antonis et al. 2004). Like in this study, dimethyl disulfide was identified as the predominant cooked off‐note contributor to thermally processed muskmelon/melon juice. Although some studies have reported a link between methionine and the formation of dimethyl disulfide, no significant relationship was found in this study (Luo et al. 2018).

6‐Methyl‐5‐hepten‐2‐one (off‐flavor), nonanal, and hexanal (characteristic flavor) are also volatile compounds derived from lipid oxidation, including auto and enzymatic oxidation (Zhang et al. 2019). 6‐Methyl‐5‐hepten‐2‐one can be derived from lycopene or other noncyclic‐tetra‐terpenoids (Lewinsohn et al. 2005). However, although hexanal was affected by heat treatment, it was not significantly related to the “aroma” results obtained in the sensory analysis. Therefore, lipid oxidation during processing or storage can cause desirable or undesirable changes in the aroma of WJ. Heat treatment causes increases in ester, acid, and ketone concentrations in WJ, while the concentration of aldehydes decreases (Wang et al. 2018). However, the results obtained in this study showed that the concentrations of both of these aroma compounds, characteristic and off‐flavor related, decreased during long‐term thermal application. Therefore, this may be related to the inactivation of lipolytic enzymes, and not the forming of oxidation‐related aroma compounds, as well as the increase in aroma loss.

### 3.2 Sensory Properties

The effects of the applied thermal treatments on the sensory quality of WJ were evaluated, and the obtained results are given in Table 1 and Figure 1. The sample treated at 79°C for 1 min had the highest scores for all the other properties, while the highest temperature–time application (95°C‐27.7 min) had the lowest overall acceptance (Table 1). The ranking test also indicated the effect of treatment time on the sensorial properties (Figure 1). All of the evaluated sensory properties of WJ decreased with increasing duration or temperature. The change tendency of the aroma material was strongly consistent with that of nonenal, nonadien‐1‐ol, hexanal, and (*E*, *Z*)‐3,6‐nonadien‐1‐ol, which are some of the aroma compounds responsible for the characteristic aroma of WJ. However, (*E*)‐2‐decenal and dimethyl disulfide did not completely reverse the change in aroma with increasing thermal treatment conditions (Figure 2). It is known that expectations about how food tastes occur are frequently based on food color, particularly red colors, which frequently exhibit persistent connections with flavor. Colors undoubtedly impact our sensory assessment of overall flavor and, thus, our food preferences (Koch and Koch 2003). The sensory analysis data confirmed this finding in the WJ (Figure 1). In another study, the results of sensory analysis showed that following thermal processing, the watermelon‐like flavor diminished with increasing temperature, the off‐flavor increased, and the color was also considerably affected (Liu et al. 2019).

**TABLE 1:** The sensory properties and aroma characteristics of watermelon juices.

Thermal treatment		Sensory properties				Aroma characteristics						
Temperature (°C)	Time (min)	Color	Odor	Taste	Overall acceptance	Cucumber	Grass*	Fruity	Floral	Fatty	Cooked	Green*
79	15.5	3.3 ± 2.7^b^	3.1 ± 2.4^bc^	3.6 ± 2.2^cde^	3.6 ± 2.2^cd^	3.7 ± 2.0^abc^	2.8 ± 2.3	3.0 ± 2.2^cd^	2.5 ± 1.7^bcd^	3.0 ± 2.4^ab^	4.7 ± 2.8^b^	3.6 ± 2.6
95	27.7	3.6 ± 2.5^b^	2.3 ± 2.2^c^	2.2 ± 1.5^e^	2.1 ± 1.2^d^	2.5 ± 2.1^bc^	2.3 ± 2.6	1.6 ± 1.0^d^	1.3 ± 2.0^d^	3.3 ± 2.2^ab^	6.8 ± 3.0^a^	2.6 ± 1.1
79	1.0	8.0 ± 2.5^a^	6.8 ± 2.2^a^	7.5 ± 1.5^a^	7.2 ± 1.2^a^	4.4 ± 2.1^a^	3.0 ± 2.6	6.6 ± 2.2^a^	4.8 ± 2.0^a^	1.6 ± 1.2^b^	1.7 ± 1.0^c^	3.3 ± 2.1
98	15.5	3.2 ± 2.6^b^	2.6 ± 2.3^c^	2.5 ± 1.9^e^	2.1 ± 1.3^d^	2.2 ± 2.2^c^	2.0 ± 2.1	1.2 ± 1.0^d^	1.2 ± 1.0^d^	4.0 ± 3.0^a^	7.1 ± 1.8^a^	1.8 ± 1.2
79	30	3.8 ± 2.1^b^	3.0 ± 2.1^c^	3.1 ± 2.4^de^	3.4 ± 2.1^cd^	3.1 ± 1.7^abc^	3.5 ± 2.0	2.9 ± 2.1^cd^	2.1 ± 1.7^cd^	2.4 ± 2.0^ab^	5.7 ± 2.3^ab^	3.4 ± 2.4
63	27.7	6.0 ± 2.2^a^	4.9 ± 2.1^ab^	5.4 ± 2.0^bc^	5.8 ± 1.9^b^	4.0 ± 2.2^abc^	2.5 ± 1.2	4.7 ± 2.9^bc^	3.5 ± 2.7^abc^	1.5 ± 1.8^b^	2.6 ± 1.9^c^	2.7 ± 1.3
95	3.3	7.7 ± 1.6^a^	4.9 ± 2.1^ab^	4.7 ± 2.4^bcd^	5.0 ± 2.0^bc^	4.7 ± 2.4^a^	3.5 ± 2.2	4.7 ± 2.7^bc^	4.3 ± 2.7^ab^	1.3 ± 0.8^b^	1.8 ± 1.0^c^	3.6 ± 2.0
63	3.3	8.0 ± 2.6^a^	6.1 ± 2.4^a^	6.3 ± 2.0^ab^	6.3 ± 2.1^ab^	4.1 ± 2.4^ab^	2.8 ± 2.4	5.8 ± 1.9^ab^	4.2 ± 2.5^ab^	1.6 ± 1.0^b^	2.4 ± 1.2^c^	3.0 ± 2.1
60	15.5	7.2 ± 1.7^a^	5.7 ± 2.3^a^	5.2 ± 2.2^bc^	5.5 ± 2.1^b^	4.3 ± 1.9^ab^	3.6 ± 2.7	4.9 ± 2.7^abc^	3.3 ± 2.3^abc^	1.1 ± 0.6^b^	2.2 ± 1.8^c^	4.0 ± 2.2

*Note:* The average values shown with different superscript letters (a–e) in the same column are different from each other (*p* < 0.05).

* There was no significant difference between the averages (*p* > 0.05).

After the heat treatments, the aroma characteristics of the WJs (cucumber, grass, fruity, floral, fatty, cooked, and greenish) were also evaluated via sensory analysis (Table 1). It was determined that heat treatment had a statistically significant effect on characteristics other than grass and greenish aromas, which can be caused by hexanal (*p* < 0.05). The greatest changes were in the “floral” and “fruity” aromas. Therefore, the change in the hexanal concentration, which occurs with heat treatment, as shown in Figure 1, was not determined via sensory analysis. WJ (79°C, 1 min), which received the highest score in terms of all evaluated sensory properties, including aroma, was the sample with the highest percentage of cucumber, fruity, and floral odors, and the lowest percentage of cooked aromas could be caused by dimethyl disulfide. It was also reported that heat treatment did not considerably change the main volatile chemicals in WJ (nonanal, (*E*)‐2‐nonenal, (*E*, *Z*)‐2,6‐nonadienal, 1‐nonanol, (*Z*)‐3‐nonen‐1‐ol, (*E*, *Z*)‐2,6‐nonadien‐1‐ol, and 6‐methyl‐5‐hepten‐2‐one), and dimethyl sulfide and methional increased in thermally treated WJ (Aboshi et al. 2020).

### 3.3 Optimum Conditions

For the optimization study, amino acid, fatty acid, HMF, lycopene, aroma, sensory, and microbiological analyses were applied to the thermally treated samples. Including all of the determined aroma compounds in optimization is not feasible, and optimization results would not be reliable in this case. Also, the inclusion of aroma substances that cannot be determined to be related to sensory properties in the optimization is not suitable for the purpose of the optimization study carried out to improve sensory properties. Therefore, the results obtained were subjected to the Pearson correlation test, and quality characteristics that showed a significant positive/negative relationship with “aroma” and/or “general impression”, the sensory analysis parameters, were included in the optimization study as constraint conditions (Table 2).

**TABLE 2:** The constraints and importance levels of the constraints of the optimization study.

Constrictions		Significance level	*R* ^2^	Adjusted *R* ^2^	Predicted *R* ^2^
Aroma	max	*****	0.9631	0.9463	0.8405
Hydroxymethyl furfural	min	*****	0.9985	0.9978	0.9957
Linolenic acid	min	**	0.9669	0.9518	0.9237
Nonadienol	max	****	0.9795	0.9702	0.9451
Nonanal	max	*****	0.9765	0.9658	0.9311
Lycopene	max	*****	0.9762	0.9654	0.9391
Leucine	max	***	0.9481	0.9407	0.9225
**Equations**					
Aroma (A): A = +20.8457 − 0.3203*T − 0.2021*t − 1.7966*10^−3^*T*t + 1.7841*10^−3^*T ^2^ + 7.5818*10^−3^*t ^2^					
Hydroxymethyl furfural (HMF): *HMF (ppb)* = 184.5697 − 5.3037*T − 1.8801*t + 0.0258*T*t + 0.0383*T ^2^ + 9.0127*10^−3^*t ^2^					
Linolenic acid (LA): LA (mA) = −94407.4639 + 2501.6645*T + 1160.3430*t + 14.0368*T*t − 13.7997*T ^2^ − 67.1022*t ^2^					
Nonadienol (ND): ND (μg/L) = −1.2579 + 0.07113*T + 0.02530*t − 2.1431*10^−3^*T*t − 2.8025*10^−3^*T ^2^ + 2.6758*10^−3^*t ^2^					
Nonanal (N): N (μg/L) = +14.8912–0.2120*T − 0.4045*t − 1.9186*10^−3^*T*t + 1.3928*10^−3^*T ^2^ + 0.0123*t ^2^					
Lycopene (Lyc): Lyc (mg/L) = +104.8859 − 2.39468*T − 0.2892*t + 8.9832*10^−5^*T*t + 0.0150*T ^2^ + 5.123*10^−3^*t ^2^					
Leucine (Leu): Leu (mg/L) = +0.9155 + 6.8318*10^−4^*T − 0.0130*t					

*Note:* T, temperature (°C); *t*, time (min). *, **, *** and **** are statistically significant.

The quality factors found to be important in relation to aroma were HMF (−0.576), leucine (0.500), lycopene (0.539), nonenal (0.524), and nonadienol (0.506). Linolenic acid was correlated with overall acceptance (−0.483). The importance levels of the constrictions were determined by considering the relationships with the “aroma” and “overall acceptance” parameters (Table 2).

Linolenic acid was reported as the most abundant unsaturated fatty acid found in watermelon, accounting for 17.5% of the total fatty acid content (Moussa et al. 2013), and the results obtained in this study showed that the linolenic acid content (12.40–40.68 mA) of WJ increased with time or temperature until a certain level was reached (Figure 2). The highest linolenic acid content was detected in WJ treated at approximately 95°C for 20 min.

The HMF content (1.11–49.67 μg/L) of WJ increased with time or temperature, especially after pasteurization at temperatures higher than 80°C (Figure 2). The significant correlation between HMF and aroma may indicate that the flavor changes resulted from the Maillard reaction. Aldehydes can be generated by various pathways, such as the oxidation of unsaturated fatty acids, Maillard reactions, Strecker degradation, degradation of bitter acids, aldol condensation, melanoid‐catalyzed oxidation of higher alcohols, and secondary autooxidation of aldehydes (Baert et al. 2012). Additionally, Maillard takes place in a medium with a pH range of 4–7, while the pH of the samples used in the present study was 3.8. Therefore, even if the HMF content, a compound produced during the Maillard reaction, is negatively correlated with the aroma score of the sample, the impact of Maillard on aroma could be related not only to aldehyde formation. Additionally, the decomposition of aldehydes by heat may have a greater effect on aroma than the formation of aldehydes via the Maillard reaction.

The leucine concentration in WJ decreased significantly with increasing heat treatment duration (Figure 2). It was reported that histidine and asparagine concentrations in grape juice decreased after pasteurization, while the total free amino acid content and total fatty acids did not change significantly (Garde‐Cerdán et al. 2007). Collateral reactions occurring throughout the production process, in which amino acids and unsaturated fatty acids are involved, cause sensory defects. When the product temperature increases, these negative effects become more significant (Garde‐Cerdán et al. 2007). However, the effect of temperature on the leucine content of WJ was not obvious.

The lycopene content (4.98–12.12 mg/L) decreased as the heat treatment time increased (Figure 2). On the other hand, lycopene content decreased with increasing temperature until reaching ~80°C but increased when higher temperatures were applied. The lycopene content can be affected by a number of factors during heat treatment, including lycopene degradation, isomerization, and more effective extraction of lycopene from the matrix. Heating can break down cell walls and release additional lycopene from the matrix (Shi et al. 2008). When the results are evaluated in general, the predominant factor is time among the treatment conditions for nonadienol, nonenal, and leucine, while the effect of temperature on HMF, linolenic acid, and lycopene content seems to be more significant.

The temperature–time combinations suggested by the central composite design of the Design–Expert program with desirability values higher than 0.844 are given in Table 3. Considering that the desirability values of the proposed heat treatment application conditions are close to each other and that they carry the lowest risk in terms of microbiology, the combination of 84°C for 1 min was chosen as the optimum heat treatment condition. When the optimum conditions were applied as pasteurization criteria, the HMF, linolenic acid, nonadienol, nonenal, aroma, lycopene, and leucin contents were 9.605 mg/L, 20.604 mg/L, 2.584 μg/L, 6.36 μg/L, 6.19, 9.004 mg/L, and 0.96 mg/L, respectively.

**TABLE 3:** Responses obtained from the optimization study and validation outputs.

	Temperature (°C)	Time (min)	HMF (μg/L)	Linolenic acid (mA)	Nonadienol (μg/L)	Nonanal (μg/L)	aroma	Lycopene (mg/L)	Leucin (mg/L)	Desirability
**1**	64.451	1.000	1.732	11.502	2.052	6.501	7.303	12.415	0.946	0.952
**2**	78.881	1.000	4.840	19.262	2.468	6.296	6.346	8.805	0.956	0.863
**3**	79.566	1.000	5.384	19.488	2.485	6.301	6.319	8.788	0.957	0.860
**4**	**83.851**	**1.000**	**9.605**	**20.604**	**2.584**	**6.360**	**6.188**	**9.004**	**0.960**	**0.844**

Validation outputs			
Properties	Responses		Error (%)
	Predicted	Actual	
Hydroxymethyl furfural (μg/L)	9.61	8.86	8.44
Linolenic acid (mA)	20.60	20.65	0.22
Nonadienol (μg/L)	2.58	2.47	4.69
Nonanal (μg/L)	6.36	6.97	8.74
Aroma	6.19	6.68	7.37
Lycopene (mg/L)	9.00	9.70	7.17
Leucin (mg/L)	0.96	1.00	3.83

*Note:* The bold values (solution) are the chosen time and temperature as optimum conditions and the predicted values of quality parameters.

### 3.4 Validation

The optimum processing conditions were applied to the WJ model to validate the obtained models. The predicted and experimental findings are given in Table 3. The results showed that error values ranging from 0.2% to 8.7% can be considered acceptable because they are not greater than 10% (Dündar et al. 2019). As a result, the optimum thermal treatment conditions were applicable, and the obtained models can be used with high assurance to predict the quality of thermally treated WJ.

## 4 Conclusion

Although the aroma compounds of WJ have been determined in some studies, the optimization of thermal treatment by including characteristic aroma compounds and some relevant quality factors, aroma precursors, was conducted for the first time to enhance WJ quality from the point of view of the consumer. The HMF, leucine, lycopene, nonenal, and nonadienol concentrations were found to be correlated with the “aroma” scores obtained via sensory analysis. Nonanal had the highest influence on the aroma of WJ among the determined aroma compounds. While all the sensory properties (color, taste, aroma, overall acceptance) were similarly affected by the heat treatment conditions, the changes in the aroma compounds (nonenal, nonadienal, hexanal, dimethyl disulfide, 6‐methyl‐5‐hepten‐one 2‐decenal) due to heat treatment differed from each other. With increasing temperature, cucumber, fruity, floral, and green odors decreased, whereas cooked and fatty odors significantly intensified. The four different temperature and time combinations were determined as a result of an optimization study with the predicted concentrations of “aroma”/“overall acceptance”‐related compounds found in WJ. To eliminate food safety concerns, 84°C and 1 min can be suggested as the pasteurization criteria for WJ (desirability value = 0.844). Optimum conditions are applicable on an industrial scale if aseptic filling with light‐proof containers and appropriate storage conditions lower than 20°C are ensured. The results obtained indicate that WJs produced on an industrial scale may be overheated. Therefore, it is possible to produce highly preferred WJs by applying lower‐intensity thermal treatments, subject to the reevaluation of the product's shelf life. In future studies, some modifications can be made in the production steps to eliminate the effects of compounds determined to have an effect on the sensory properties of WJ. These modifications, such as resin treatments, may be designed to selectively degrade or remove aroma precursors from the matrix, considering the side effects on quality properties. Thus, the production of WJ can increase, and watermelon consumption can increase.

## Author Contributions

**Burcu Dundar Kirit:** conceptualization (equal), formal analysis (lead), writing – original draft (lead), writing – review and editing (equal). **Erdal Ağçam:** methodology (equal), writing – review and editing (equal). **Asiye Akyildiz:** methodology (equal), supervision (lead), writing – review and editing (equal).

## Ethics Statement

The appropriate protocols such as no coercion to participate, full disclosure of study requirements and risks, verbal consent of participants, no release of participant data without their knowledge, and ability to withdraw from the study at any time for protecting the rights of panelists were applied in the sensory evaluation.

## Conflicts of Interest

The authors declare no conflicts of interest.

## Supporting information


File S1

File S2

File S3

File S4

File S5

File S6

File S7

## Data Availability

The authors declare that the data supporting the findings of this study are available within the paper and its Files S1–S7. The raw data files needed in another format are available from the corresponding author upon reasonable request.
