# Supplementary material for: An Aroma Precursor‐Based Approach to Improving the Sensory Quality of Thermally Treated Watermelon Juice
Source: Food Sci Nutr. 2025 Jun 13;13(6):e70342. doi: 10.1002/fsn3.70342 (PMC12163749; doi:10.1002/fsn3.70342)
Supplement: Supplementary file 1 — File S1 [file FSN3-13-e70342-s006.docx]

Watermelon

Washing

Peeling

Seed removal

Mashing

**Optimization**

(Pasteurization)

Aseptic filling

(

Sieving

Watermelon juice

pH adjusting

Slicing

*Control 1*

**Analyses**

(

Supplementary Material 1. Production steps of watermelon juice
