# Supplementary material for: An Aroma Precursor‐Based Approach to Improving the Sensory Quality of Thermally Treated Watermelon Juice
Source: Food Sci Nutr. 2025 Jun 13;13(6):e70342. doi: 10.1002/fsn3.70342 (PMC12163749; doi:10.1002/fsn3.70342)
Supplement: Supplementary file 2 — File S2 [file FSN3-13-e70342-s004.docx]

Supplementary Material 2. Heat exchanger: Magnetic stirrer (1), Peristaltic pump (2), Stainless steel heat exchanger (3), Cooling chamber (4) (Agcam, Akyıldız, & Akdemir Evrendilek, 2014).
