# Supplementary material for: An Aroma Precursor‐Based Approach to Improving the Sensory Quality of Thermally Treated Watermelon Juice
Source: Food Sci Nutr. 2025 Jun 13;13(6):e70342. doi: 10.1002/fsn3.70342 (PMC12163749; doi:10.1002/fsn3.70342)
Supplement: Supplementary file 3 — File S3 [file FSN3-13-e70342-s007.docx]

Supplementary Material 3. Thermal treatments applied for optimization study

| **Run** | **Temperature (°C)** | **Time (min)** |
| --- | --- | --- |
| 1 | 79 | 15.5 |
| 2 | 79 | 1.0 |
| 3 | 79 | 15.5 |
| 4 | 79 | 30.0 |
| 5 | 79 | 15.5 |
| 6 | 79 | 1.0 |
| 7 | 63 | 27.7 |
| 8 | 60 | 15.5 |
| 9 | 63 | 3.3 |
| 10 | 95 | 3.3 |
| 11 | 79 | 15.5 |
| 12 | 79 | 30.0 |
| 13 | 95 | 27.7 |
| 14 | 60 | 15.5 |
| 15 | 98 | 15.5 |
| 16 | 79 | 15.5 |
| 17 | 98 | 15.5 |
