# Supplementary material for: An Aroma Precursor‐Based Approach to Improving the Sensory Quality of Thermally Treated Watermelon Juice
Source: Food Sci Nutr. 2025 Jun 13;13(6):e70342. doi: 10.1002/fsn3.70342 (PMC12163749; doi:10.1002/fsn3.70342)
Supplement: Supplementary file 4 — File S4 [file FSN3-13-e70342-s005.docx]

Supplementary Material 4.a. The HPLC chromatogram of amino acids

Supplementary Material 4.b. The HPLC chromatogram of lycopene

Supplementary Material 4.c. The HPLC chromatogram of hydroxymethyl furfural (HMF)

4

1

5

3

2

Supplementary Material 4.d. The GC-MS chromatogram of aroma compounds

1: hexanal, 2: 6-methyl-5-hepten-2-one, 3: (*E*)-2-nonenal; 4: (*E*,*Z*)-2,6-nonadienal, ,5: (*E*,*Z*)-3,6-nonadien-1-ol,

5

4

3

2

1

Supplementary Material 4.e. The GC-MS chromatogram of fatty acids

1: palmitic acid, 2: stearic acid, 3: oleic acid, 4: linoleic acid, 5: linolenic acid
