# Supplementary material for: An Aroma Precursor‐Based Approach to Improving the Sensory Quality of Thermally Treated Watermelon Juice
Source: Food Sci Nutr. 2025 Jun 13;13(6):e70342. doi: 10.1002/fsn3.70342 (PMC12163749; doi:10.1002/fsn3.70342)
Supplement: Supplementary file 5 — File S5 [file FSN3-13-e70342-s001.docx]

Supplementary Material 5. Retention indices and odor thresholds of aroma compounds

| No | **RI** | **OT (µg/kg)** | **Aroma compounds** |
| --- | --- | --- | --- |
|  |  |  |  |
| 1 | 1545 | 0.1^[1]^ | (*Z*)-2-Nonenal |
| 2 | 1092 | 4.5^[2]^ | Hexanal |
| 3 | 1244 | 0.1^[3]^ | (*E*)-2-Hexenal |
| 4 | 1244 | 0.12^[4]^ | (*E*)-2-Decenal |
| 5 | 1577 | 0.09^[5]^ | (*E*)-2-Nonenal |
| 6 | 1627 | 0.2^[6]^ | (*E*,*Z*)-2,6-Nonadienal |
| 7 | 1325 | 0.7^[7]^ | Octanal |
| 8 | 1436 | 1.0^[8]^ | Nonanal |
| 9 | 1468 | 0.12^[9]^ | (*E*)-2-Octenal |
| 10 | 1541 | 0.18^[10]^ | Decanal |
| 11 | 1867 | 0.5^[11]^ | 4-Oxononanal |
| 12 | 1386 | 4.0^[12]^ | 1-Hexanol |
| 13 | 1595 | 14.2^[13]^ | 1-Octanol |
| 14 | 1415 | 0.1^[14]^ | Dimethyl trisulfide |
| 15 | 1265 | - | 2-pentil-furan |
| 16 | 1372 | - | 6-methyl-5-hepten-2-on |
| 17 | 1990 | - | (*E*)-beta-iyonon |

RI: Kovats indices (calculated), OT: Odor threshold values (literature)

1. Shimoda, M., Shigematsu, H., Shiratsuchi, H., & Osajima, Y. (1995). Comparison of the odor concentrates by SDE and adsorptive column method from green tea infusion. Journal of Agricultural and Food Chemistry, 43(6), 1616–1620. https://doi.org/10.1021/jf00054a039
2. Mulders, E. J. (1973). The odour of white bread: IV. Quantitative determination of constituents in the vapour and their odour values. Zeitschrift für Lebensmittel-Untersuchung und Forschung, 151, 310–317.
3. Karagül-Yüceer, Y., Vlahovich, K. N., Drake, M., & Cadwallader, K. R. (2003). Characteristic aroma components of rennet casein. Journal of Agricultural and Food Chemistry, 51(23), 6797–6801.
4. Larsen, M., & Poll, L. (1990). Geruchschwellen einiger wichtiger Aromastoffe der Himbeeren. Zeitschrift für Lebensmittel-Untersuchung und Forschung, 191, 129–131.
5. Blank, I., Sen, A., & Grosch, W. (1992). Sensory study on the character-impact flavour compounds of dill herb (Anethum graveolens L.). Food Chemistry, 43(5), 337–343.
6. Guadagni, D. G., Buttery, R. G., & Harris, J. (1966). Odour intensities of hop oil components. Journal of the Science of Food and Agriculture, 17(3), 142–144.
7. Buttery, R. G., & Ling, L. C. (1995). Volatile flavor components of corn tortillas and related products. Journal of Agricultural and Food Chemistry, 43(7), 1878–1882.
8. Patton, S. (1964). Flavor thresholds of volatile fatty acids. Journal of Food Science, 29(5), 679–680.
9. Schnabel, K. O., Belitz, H. D., & von Ranson, C. (1988). Investigations on the structure-activity-relationships of odorous substances. Communication I. Detection thresholds and odour qualities of aliphatic and alicyclic compounds containing oxygen functions. Zeitschrift für Lebensmittel-Untersuchung und Forschung, 187, 215–223.
10. Buttery, R. G., Black, D. R., Guadagni, D. G., Ling, L. C., Connolly, G., & Teranishi, R. (1974). California bay oil. I. Constituents, odor properties. Journal of Agricultural and Food Chemistry, 22(5), 773–777.
11. Amoore, J. E., & Venstrom, D. (1966). Sensory analysis of odor qualities in terms of the stereochemical theory. Journal of Food Science, 31(1), 118–128.
12. Pyysalo, T., Suihko, M., & Honkanen, E. (1977). Odour thresholds of the major volatiles identified in cloudberry (Rubus chamaemorus L.) and arctic bramble (Rubus arcticus L.). Lebensmittel-Wissenschaft und -Technologie, 10, 36–39.
13. Adedeji, J., Hartman, T. G., Rosen, R. T., & Ho, C. T. (1991). Free and glycosidically bound aroma compounds in hog plum (Spondias mombins L.). Journal of Agricultural and Food Chemistry, 39(8), 1494–1497.
14. Moio, L., Langlois, D., Etievant, P. X., & Addeo, F. (1993). Powerful odorants in water buffalo and bovine Mozzarella cheese by use of extract dilution sniffing analysis. Italian Journal of Food Science, 5(3), 227–237.
