# Supplementary material for: An Aroma Precursor‐Based Approach to Improving the Sensory Quality of Thermally Treated Watermelon Juice
Source: Food Sci Nutr. 2025 Jun 13;13(6):e70342. doi: 10.1002/fsn3.70342 (PMC12163749; doi:10.1002/fsn3.70342)
Supplement: Supplementary file 6 — File S6 [file FSN3-13-e70342-s002.docx]

Supplementary Material 6. Sensory evaluation forms of watermelon juice samples

**Name-Surname: Date:**

**Sample No:**

In the scales given below, the strength of the descriptors increases from left to right. Evaluate the odor characteristics of the samples by placing an "**X**" mark where you deem appropriate on the scale.

weak strong

Cucumber

Grass

Fruity

Floral

Oily

Cooked

Green

The desirability of sensory characteristics in the scales given below increases from left to right. Evaluate the sensory characteristics of the samples by placing an "**X**" on the scale.

weak strong

Color

Aroma

Taste

Overall Acceptance
