# Supplementary material for: An Aroma Precursor‐Based Approach to Improving the Sensory Quality of Thermally Treated Watermelon Juice
Source: Food Sci Nutr. 2025 Jun 13;13(6):e70342. doi: 10.1002/fsn3.70342 (PMC12163749; doi:10.1002/fsn3.70342)
Supplement: Supplementary file 7 — File S7 [file FSN3-13-e70342-s003.docx]

Supplementary Information 7. The results of pH and titratable acidity analysis in watermelon juices

|  |  |  |  |  |
| --- | --- | --- | --- | --- |
| **Sample** | **Temperature (°C)** | **Time (min)** | **pH** | **Titratable acidity (%ca)** |
| **Control** | - | - | 3.81^g^ | 0.474^a^ |
| **1** | 79 | 15.5 | 3.88^b^ | 0.403^def^ |
| **2** | 79 | 1 | 3.87^c^ | 0.397^def^ |
| **3** | 79 | 15.5 | 3.86^c^ | 0.433^bcd^ |
| **4** | 79 | 30 | 3.85^de^ | 0.314^gh^ |
| **5** | 79 | 15.5 | 3.83^f^ | 0.319^gh^ |
| **6** | 79 | 1 | 3.85^de^ | 0.376^f^ |
| **7** | 63 | 27.7 | 3.86^cd^ | 0.286^h^ |
| **8** | 60 | 15.5 | 3.97^a^ | 0.391^ef^ |
| **9** | 63 | 3.3 | 3.85^de^ | 0.332^g^ |
| **10** | 95 | 3.3 | 3.85^de^ | 0.378^f^ |
| **11** | 79 | 15.5 | 3.85^de^ | 0.303^gh^ |
| **12** | 79 | 30 | 3.83^f^ | 0.334^g^ |
| **13** | 95 | 27.7 | 3.85^de^ | 0.380^f^ |
| **14** | 60 | 15.5 | 3.89^b^ | 0.305^gh^ |
| **15** | 98 | 15.5 | 3.85^de^ | 0.410^def^ |
| **16** | 79 | 15.5 | 3.84^ef^ | 0.450^abc^ |
| **17** | 98 | 15.5 | 3.84^ef^ | 0.459^ab^ |

^a-j^: Average values shown with different letters in the same column are different from each other (*p*˂0.05). ca: citric acid
